# Synthesis of a novel porous organic polymer containing triazine and cyclohexanone rings as an efficient methyl red adsorbent from aqueous solutions

**DOI:** 10.1038/s41598-023-40274-7

**Published:** 2023-08-10

**Authors:** Javad Ghanbari, Akbar Mobinikhaledi

**Affiliations:** 1 Department of Chemistry, Faculty of Science, Arak University, Arak, 38156-88138 Iran; 2 Institute of Nanosciences and Nanotechnology, Arak University, Arak, Iran

**Keywords:** Chemistry, Materials science

## Abstract

In this research, a new porous organic polymer based on triazine and cyclohexanone rings was synthesized via Schiff base condensation, and its performance as an adsorbent for the removal of Methyl Red dye from aqueous solution was investigated. The synthesized polymer was characterized by FT-IR, XRD, SEM, EDS, TEM, TGA, and BET analyses. Five important parameters of pH (4–10), contact time (10–120 min), adsorbent dose (5–10 mg), initial dye concentration (10–70 mg/L), and temperature (25–45 °C) were investigated to optimize the adsorption conditions. Solution pH of 4, contact time of 80 min, adsorbent dose of 8 mg, initial dye concentration of 50 mg/L, and temperature of 45 °C were obtained as the best conditions for the adsorption of methyl red dye. Two widely used Langmuir and Freundlich models were employed to investigate the adsorption isotherm, and the obtained data showed that the adsorption process follows the Langmuir isotherm with a correlation coefficient (R^2^ = 0.9784) which indicates monolayer adsorption. The achieved maximum adsorption capacity was 178.57 mg/g. Also, the results of kinetic studies indicate that the adsorption process follows the pseudo-second-order kinetic, which suggests that chemical interactions play an important role in dye removal. Furthermore, the results showed that the adsorption process of methyl red dye by polymer is endothermic.

## Introduction

Nowadays, with the expansion of diverse industries such as textile, pharmaceutical, chemical, petrochemical, etc., the contamination of water sources has increased^1–5^. Some of the most consequential pollutants in wastewater are heavy metals, pesticides, pathogenic agents, artificial dyes, and detergents^6–10^. One of the biggest sources of water pollution is the dyeing industry with a share of about 17–20%^11^. Discharging colored and non-colored wastes from different industries such as textile, paper making, cosmetics, agriculture, plastic, pharmaceuticals, and leather create severe environmental problems^12–14^. Among the various industries, the textile industry has made great progress in recent years. Recent studies show that over 10,000 tons of dyes are used annually to produce 40 million tons of textiles around the world. About 5000 tons of these colors and 3600 tons of various wastes with a high concentration of colors are dumped into different water sources^15,16^. Various dyes such as thymol blue (TB), rhodamine B (RhB), Congo red (CR), methylene blue (MB), methyl orange (MO), and methyl red (MR) are used in the textile industry. Methyl red is among the most widely used of these dyes. These dyes are resistant to biological decomposition processes^17^. The presence of dyes in wastewater prevents the penetration of sunlight into the water, and the speed of photokinetic processes in surface waters decreases^18,19^. In addition, most textile dyes have an organic origin and are mainly prepared from diazo, phthalocyanine, and anthraquinone salts which have a benzene ring^20^. Among these dyes, azo dyes are one of the most important and widely used dyes, which make up about 90% of synthetic organic dyes^21^. Also, this group of dyes is one of the most hazardous sources of pollution in aquatic environments^22,23^. The effective removal of this group of dyes has become a global challenge for researchers due to their toxicity and high level of pollution. Methyl red dye is one of the azo dyes, which is used as a pH indicator in the laboratory^24^. Also due to the presence of N=N bond between two aromatic rings which stabilizes its chemical properties and as a result, it increases the power of high color stabilization and fading of low color and its high consumption in dyeing and printing industries^25^. Despite having these very valuable and effective properties, swallowing or inhaling of MR can cause sensitivity and irritation to the skin, eyes, gastrointestinal tract, or throat^26,27^. Also, this compound is carcinogenic and mutagenic and does not decompose easily in environment due to the presence of benzene rings^28,29^. Therefore, considering the harmful effects of MR on living things and plants, it is necessary to treat the wastewater containing this substance before discharging it into the environment and water resources. So far, several chemical, biological, and physical techniques, such as ion exchange, reverse osmosis oxidation, ozonation, flocculation, electrolysis, coagulation, photocatalysis, sedimentation, biodegradation, and membrane filtration have been reported by researchers to remove MR and other dyes from wastewater^30–33^. However, some of these methods have some disadvantages such as complex and expensive technologies, lack of high efficiency of dye removal, and production of significant amounts of sludge, which leads to other environmental problems. The existence of such problems has always prompted researchers to look for new methods in this field. Recently, the surface adsorption method has received considerable attention due to its simplicity, practicality, cheapness, and high efficiency^34–38^. Until now, various adsorbents such as activated carbon, modified zeolites, clay, bismuth oxychloride (BiOCl), silica, char, and biomass have been used to remove MR from aqueous environments^39–44^. Nowadays, porous materials have attracted the special attention of researchers as very useful and efficient adsorbents. One of these porous adsorbents is porous organic polymers (POPs). POPs are an important class of porous materials that are formed through strong covalent interactions between organic monomers containing light elements such as carbon and oxygen, sulfur, nitrogen, boron, and phosphorus^45,46^. Due to the progress of science and technology, various POPs such as conjugated microporous polymers (CMPs), covalent organic frameworks (COFs), hyper-crosslinked polymers (HCPs), covalent triazine frameworks (CTFs), polymers of intrinsic microporosity (PIMs), porous aromatic frameworks (PAFs) and metal–organic frameworks (MOFs) have been synthesized^47–49^. POPs are superior to other porous materials due to having useful properties such as high porosity, low density, thermal and chemical stability, large specific surface area, outstanding designability, excellent photoelectron ability, robustness, and tuneable structures^50–52^. The mentioned properties have caused POPs to have many applications such as gas separation, heterogeneous catalysis, drug delivery, energy, and gas storage, supercapacitors, water treatment, sensors, and proton conductors^53,54^. POPs with their unique properties have become a promising candidates as high-performance adsorbents for the removal of pollutants from aqueous media. With attention to the great potential of this class of polymers as adsorbents, so far little research has been reported on the removal of methyl red dye from aqueous environments by POPs. According to these considerations, herein we report the synthesis of a new triazine and cyclohexanone-based porous organic polymer (TC-POP) with high oxygen and nitrogen content by Schiff’s base condensation and examine its effectiveness in removing methyl red dye from aqueous media.

## Materials and methods

### Materials

Cyanuric chloride (99%), p-hydroxybenzaldehyde (98%), sodium hydroxide (NaOH) (99%), cyclohexanone (99.8%), 4-nitrobenzaldehyde (98%), potassium hydroxide (KOH) (≥ 85%), sodium sulfide (Na_2_S) (≥ 98%), sodium bicarbonate (NaHCO_3_) (99%), methyl red (MR) (≥ 99%), chlorhydric acid (HCl) (37%), acetone (99%), ethanol (99%), ethyl acetate (99.5%), n-hexane (99%), methanol (99%), n-butanol (99.5%), o-dichlorobenzene (≥ 99%), acetic acid (100%), *N*,*N*-dimethylformamide (DMF) (≥ 99.8%), dichloromethane (**≥ **99.5%), and tetrahydrofuran (THF) (**≥ **99.8%) were prepared from Aldrich and Merck companies and used without further purification.

### Characterization techniques

Fourier transform infrared (FT-IR) spectra was registered on an ALPHA-Bruker spectrometer from 400 to 4000 cm^−1^, using KBr pellet. ^1^H-NMR (300 MHz) and ^13^C-NMR (75 MHz) were carried out in CDCl_3_ on a Bruker-Avance spectrometer. The thermogravimetry analysis (TGA) was performed on a Q600 TA instrument under an argon atmosphere at 25–800 °C. X-ray diffraction (XRD) data were examined by X’Pert- MPD PRO-PW3040/60 instrument. The surface morphology and atomic distribution were obtained by field emission scanning electron microscope (FE-SEM) brand TESCAN (MIRA 3 LMU). Zeiss EM900 microscope was used for Transmission electron microscopy (TEM) analysis. The surface area, pore volume, and pore size distribution were characterized on a BELSORP MINI II system at 77 K using N_2_ as the adsorbate. The UV–Visible (UV–Vis) spectra of the methyl red dye solutions were recorded using a UV-2550 spectrophotometer.

### Synthesis of 2,4,6-tris-(4-formylphenoxy)1,3,5-triazine (TFPT) (3)

This triazine compound **(3)** was preapred according to our earlier method^55^. Briefly, a solution of p-hydroxybenzaldehyde **(1)** (1.89 g, 15.5 mmol) and NaOH (0.62 g, 15.5 mmol) in acetone and water (40 mL, 1:1 v/v) was prepared over a magnetic stirrer at 0 °C. Then, a solution of cyanuric chloride **(2)** (0.92 g, 5.0 mmol) in acetone (20 mL) was added dropwise over 60 min. Afterward, the obtained solution was stirred for 2 h at room temperature and refluxed for 10 h. The reaction mixture was then poured into 50 mL water to precipitate the desired white product. The precipitate was filtered and washed with distilled water and ethanol three times and recrystallized from ethyl acetate to give pure product, which then dried at 70 °C under a vacuum for 12 h.

### 6-bis(4-nitrobenzylidene)cyclohexanone (6)

According to the reported method^56^, a mixture of cyclohexanone **(4)** (0.49 g, 5 mmol), 4-nitrobenzaldehyde **(5)** (1.51 g, 10 mmol), KOH (0.11 g, 2 mmol), and ethanol (10 mL) was stirred using a magnetic stirrer at 40 °C. After completion of the reaction (monitored by TLC, ethyl acetate/n-hexane 1:1 v/v), the reaction mixture was cooled to room temperature, and the obtained yellow solid product was filtered and washed thoroughly with water and dried, then recrystallized from ethanol to give the pure product.

### 6-bis(4-aminobenzylidene)cyclohexanone (7)

A mixture of Na_2_S (0.80 g, 10.2 mmol) and NaHCO_3_ (0.35 g, 4.16 mmol) was dissolved in 5 mL of water. Then, methanol (10 mL) was added, and the reaction mixture stirred for 30 min at room temperature. The white precipitate was filtered and the filtered solution was added to a mixture of 2,6-bis(4-nitrobenzylidene)cyclohexanone **(6)** (0.26 g, 0.714 mmol) and 15 mL methanol, and stirred for 3 h under reflux conditions. The mixture was concentrated using a rotary evaporator and the residue poured into water, the red product was filtered, washed well with water, and finally dried^57^.

### Synthesis of triazine and cyclohexanone-based porous organic polymer (TC-POP) (8)

A mixture of 2,4,6-tris-(4-formylphenoxy)1,3,5-triazine (TFPT) **(3)** (0.178 g, 0.4 mmol) and 2,6-bis(4-aminobenzylidene)cyclohexanone **(7)** (0.183 g, 0.6 mmol) was poured in a 10 mL pyrex tube. Then, 2 mL solution of n-butanol/o-dichlorobenzene (1:1 v/v) was added to the mixture and sonicated for 30 min. Subsequently, acetic acid (6 M, 400 μL) was added and the tube sealed and heated at 85 °C for 7 days. The reaction mixture was cooled to room temperature, the resulting brown precipitate was collected by centrifugation and washed several times with *N*,*N*-dimethylformamide, dichloromethane, ethyl acetate, acetone, and tetrahydrofuran to eliminate the ensnared guest molecules. Eventually, the product was dried at 80 °C for 24 h under a vacuum.

### Methyl red adsorption experiments

A stock solution (1000 ppm) of methyl red in deionized water was prepared, and the desired solutions were also prepared by dilution of this stock solution. To investigate the removal of methyl red by POP, 8 mg of TC-POP was poured into 10 mL of 50 mg/L MR solutions and stirred at room temperature for 24 h by magnetic stirring The adsorbent was separated by centrifuge and the residual concentration of MR was determined by UV–vis spectrophotometer at λ_max_ = 430 nm. Then, the effect pH (adjusted with 0.1 M of NaOH and 0.1 M HCl solutions), contact time, initial dye concentration, the amount of adsorbent, and temperature were examined in adsorption process. The adsorption follows Eqs. (1) and (2).

1 $${Q}_{e}=\frac{{(C}_{0}-{C}_{e})V}{m}$$

2 $${\%R}_{e}=\frac{{(C}_{0}-{C}_{e})}{{C}_{0}}\times 100$$

where C_0_, C_e_ (mg/L), m (g), and V (L) refer to the initial and equilibrium concentration of methyl red, the mass of adsorbent and the volume of solution, respectively.

## Results and discussion

### Characterization of the TC-POP

**Figure 1:**
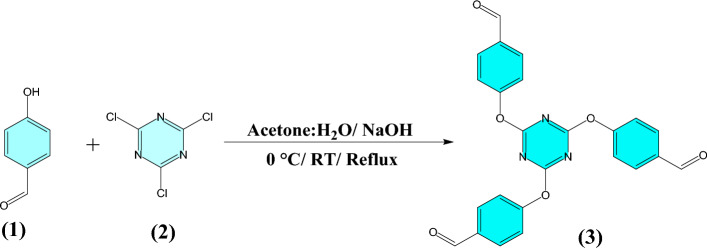
Synthesis of 2,4,6-tris-(4-formylphenoxy)1,3,5-triazine (TFPT).

**Figure 2:**
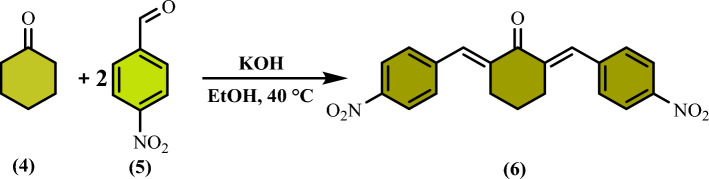
Synthesis of 2,6-bis(4-nitrobenzylidene)cyclohexanone.

**Figure 3:**
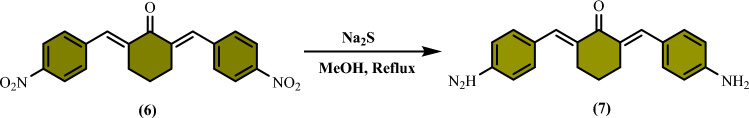
Synthesis of 2,6-bis(4-aminobenzylidene)cyclohexanone.

Initially, the compound 2,4,6-tris-(4-formylphenoxy)1,3,5-triazine (TFPT) **(3)** was prepared by the reaction of p-hydroxybenzaldehyde **(1)** and cyanuric chloride **(2)** under alkaline condition (Fig. 1). Then, compound 2,6-bis(4-aminobenzylidene)cyclohexanone **(7)** was synthesized in two steps. In the first step, 2,6-bis(4-nitrobenzylidene)cyclohexanone **(6)** was prepared through Knoevenagel condensation between one equimolar cyclohexanone **(4)** and two equimolar 4-nitrobenzaldehyde **(5)** (Fig. 2). Subsequently, compound 2,6-bis(4-nitrobenzylidene)cyclohexanone **(6)** was reduced by using Na_2_S to 2,6-bis(4-aminobenzylidene)cyclohexanone **(7)** (Fig. 3). Compounds 3 and 7 were characterized by FT-IR and NMR data (Fig. S1–S7, Supporting Information).

**Figure 4:**
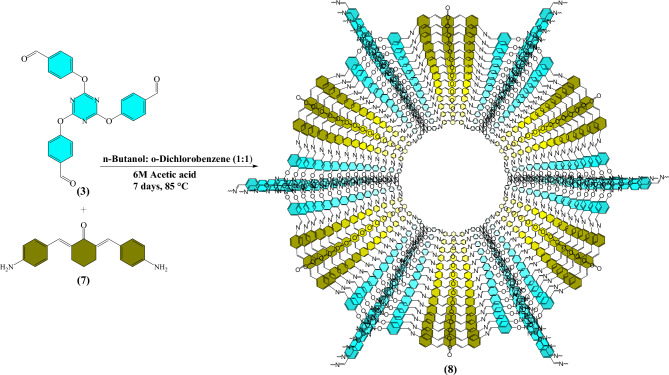
The synthetic pathway of triazine and cyclohexanone-based porous organic polymer (TC-POP).

The triazine and cyclohexanone-based porous organic polymer (TC-POP) **(8)** was then synthesized via a Schiff-base condensation of 2,4,6-tris-(4-formylphenoxy)1,3,5-triazine **(3)** and 2,6-bis(4-aminobenzylidene)cyclohexanone **(7)** as presented in Fig. 4.

**Figure 5:**
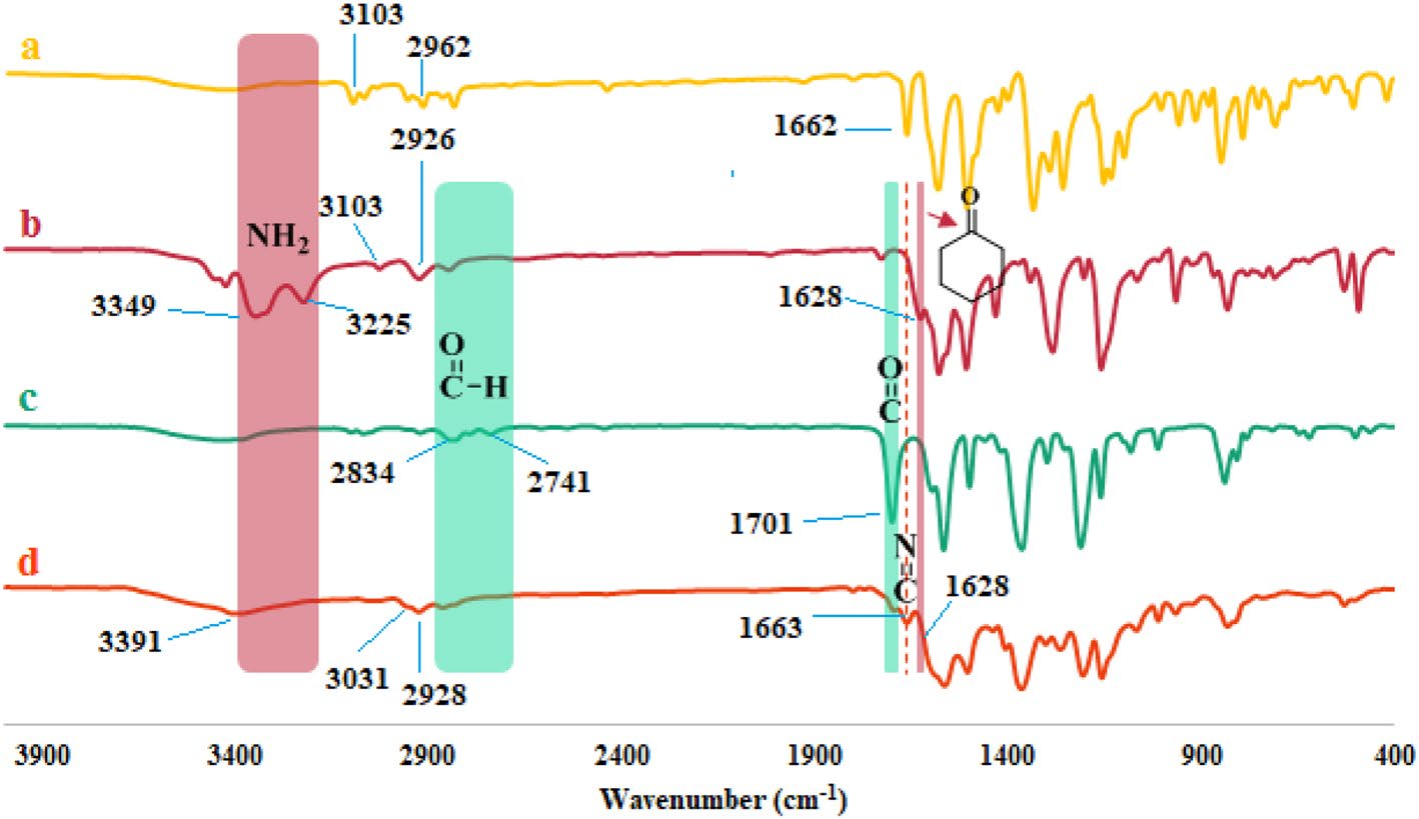
FT-IR spectra of (**a**) 2,6-bis(4-nitrobenzylidene)cyclohexanone, (**b**) 2,6-bis(4-aminobenzylidene)cyclohexanone, (**c**) tris-aldehyde (TFPT), and (**d**) triazine and cyclohexanone-based porous organic polymer.

The structure of TC-POP was characterized using different analytical techniques. The Fourier transform infrared (FT-IR) spectrum of 2,6-bis(4-nitrobenzylidene)cyclohexanone **(6)**, 2,6-bis(4-aminobenzylidene)cyclohexanone **(7)**, 2,4,6-tris-(4-formylphenoxy)1,3,5-triazine (TFPT) **(3)** and TC-POP **(8)** in the region of 400–4000 cm^−1^ is shown in Fig. 5a–d. The appearance of a new absorption band in the area of 1663 cm^−1^, which is related to the stretching vibration of the imine (C=N) bond, and also, the disappearance of vibration bands related to C=O (1701 cm^−1^) and C–H (2741, 2834 cm^−1^) bonds of 2,4,6-tris-(4-formylphenoxy)1,3,5-triazine (TFPT) **(3)** and N–H (3225, 3349 cm^−1^) bond of 2,6-bis(4-aminobenzylidene)cyclohexanone **(7)**, indicate the successful synthesis of TC-POP.

**Figure 6:**
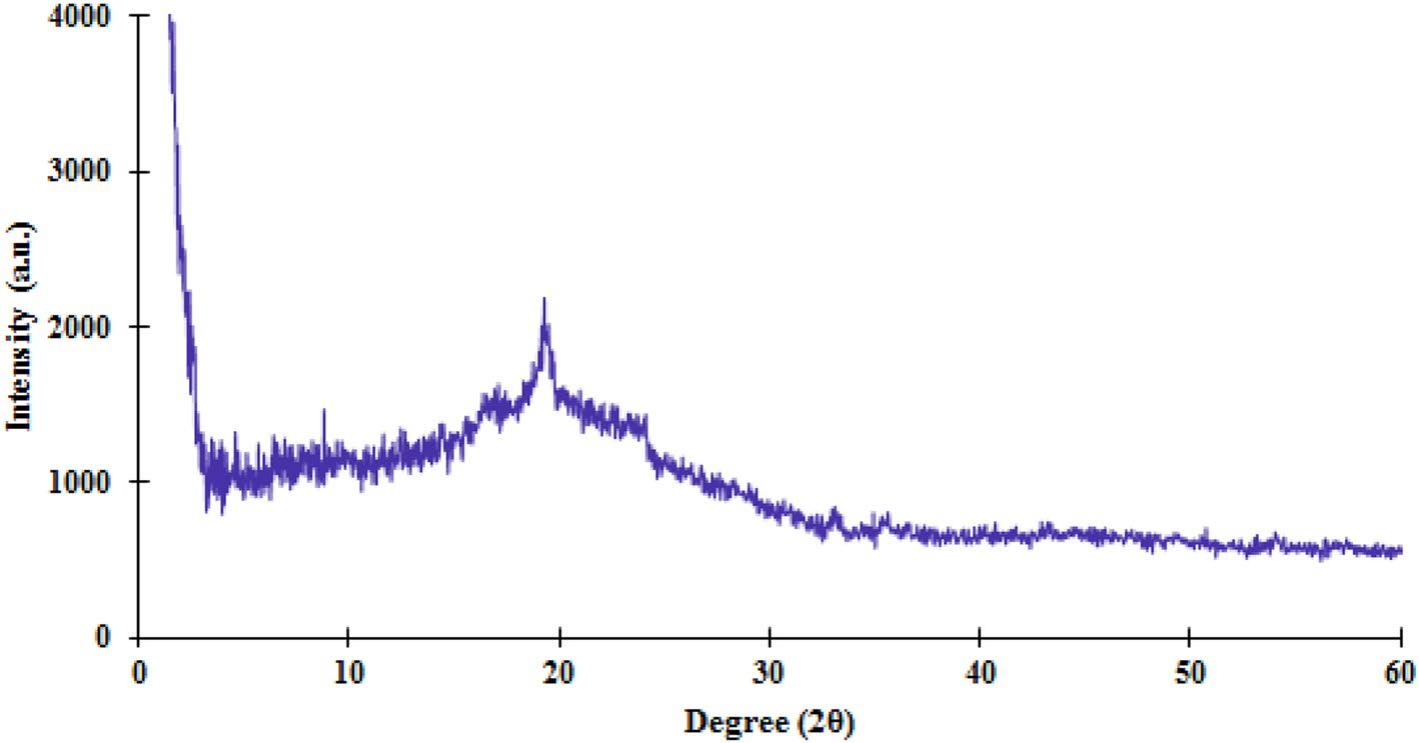
The XRD spectra of the triazine and cyclohexanone-based porous organic polymer.

X-ray diffraction (PXRD) pattern was used to investigate the crystalline structure of synthesized TC-POP. As shown in Fig. 6, due to the appearance of a peak at 2θ = 20° and according to PXRD diagram, it can be concluded that the synthetic polymer has an amorphous structure, which is one of the characteristics of POPs.

**Figure 7:**
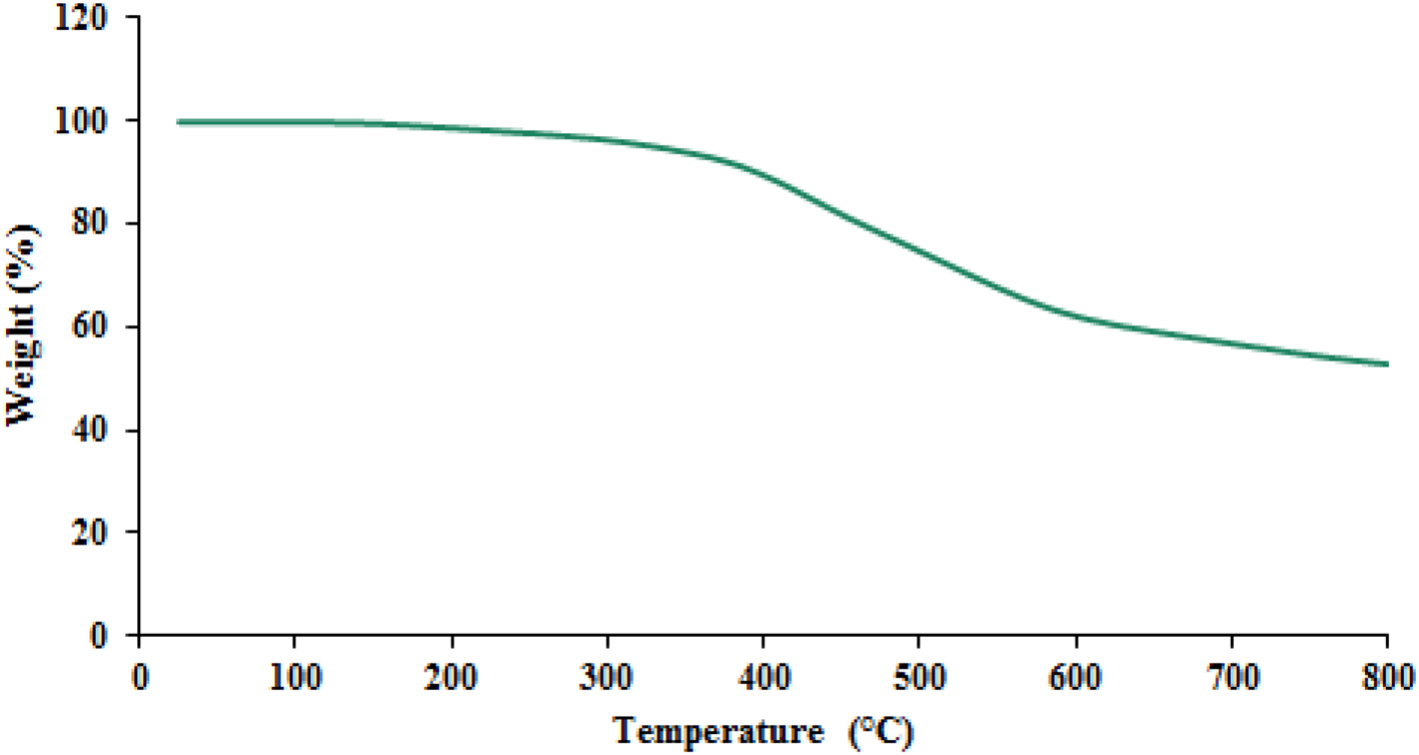
The TGA curve of TC-POP.

The thermal stability of TC-POP was investigated by thermogravimetric analysis (TGA) in the temperature limit of 25–800 °C and under an argon atmosphere. As can be seen in the TGA curve (Fig. 7), the synthetic polymer shows a good resistance to heat up to 400 °C due to strong covalent bonds between its constituent monomers. Below 250 °C, there is a mass reduction, which is related to the removal of the adsorbed moisture (water), unreacted monomers, and organic solvents trapped in TC-POP.

**Figure 8:**
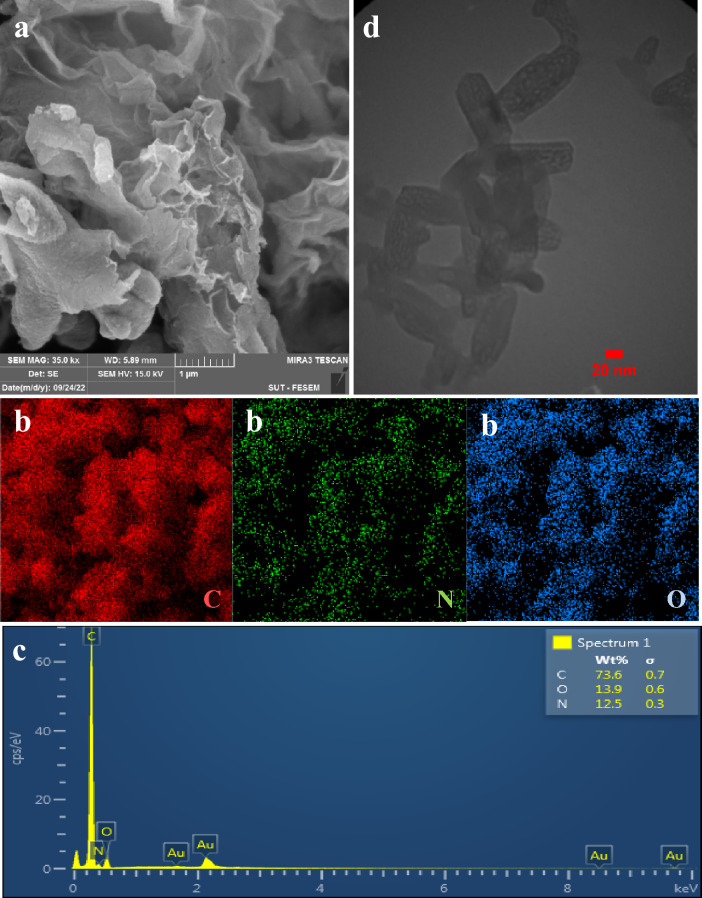
SEM images (**a**), EDX elemental mapping of C, N, O (**b**), EDX spectrum (**c**), and TEM images (**d**) of TC-POP.

Scanning electron microscope (SEM) and transmission electron microscope (TEM) were used to investigate the morphology and structure porosity of the synthesized TC-POP (Fig. 8). According to the SEM image in Fig. 8a, the morphology of the prepared polymer is layered and similar to the cabbage plant. The elemental mapping analysis of POP is shown in Fig. 8b, which shows the presence and dispersion of C (red), N (green), and O (blue) elements uniformly on the surface of the material, and also, the energy dispersive X-ray (EDX) spectrum of TC-POP (Fig. 8c) shows the elements carbon, nitrogen, and oxygen, which confirm the successful synthesis of the polymer. The transmission electron microscopy (TEM) image of TC-POP is shown in Fig. 8d, which proves the existence of porosity in the structure of the polymer.

**Figure 9:**
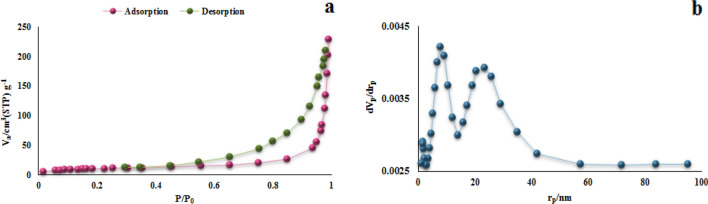
The N_2_ adsorption–desorption isotherm (**a**) and pore size distribution of TC-POP (**b**).

The surface area and pore characteristics of the polymer structure were determined by experiment and analyze nitrogen adsorption/desorption at a temperature of 77 K. As shown in Fig. 9a, the resulting polymer shows nitrogen gas sorption isotherm type IV according to the International Union of Pure and Applied Chemistry classification. The Brunauer–Emmett–Teller surface area (S_BET_) and the total pore volume of the TC-POP were calculated to be 108.27 m^2^ g^−1^ and 0.1965 cm^3^ g^−1^, respectively. In addition, the pore size dispensation is shown in Fig. 9b, which was calculated based on the Barret–Joyner–Halenda (BJH) technique. The micropore size peaked in the range of 1.85, 7.83, and 24.81 nm, which the size of most pores being around 7.83 nm.

### Methyl red adsorption studies

After the synthesis of the polymer, the adsorption of methyl red dye was studied under optimal conditions to examine its performance as an adsorbent. In order to optimize the adsorption conditions, five important parameters of pH, contact time, adsorbent dose, initial dye concentration, and temperature were investigated.

### Effect of pH

**Figure 10:**
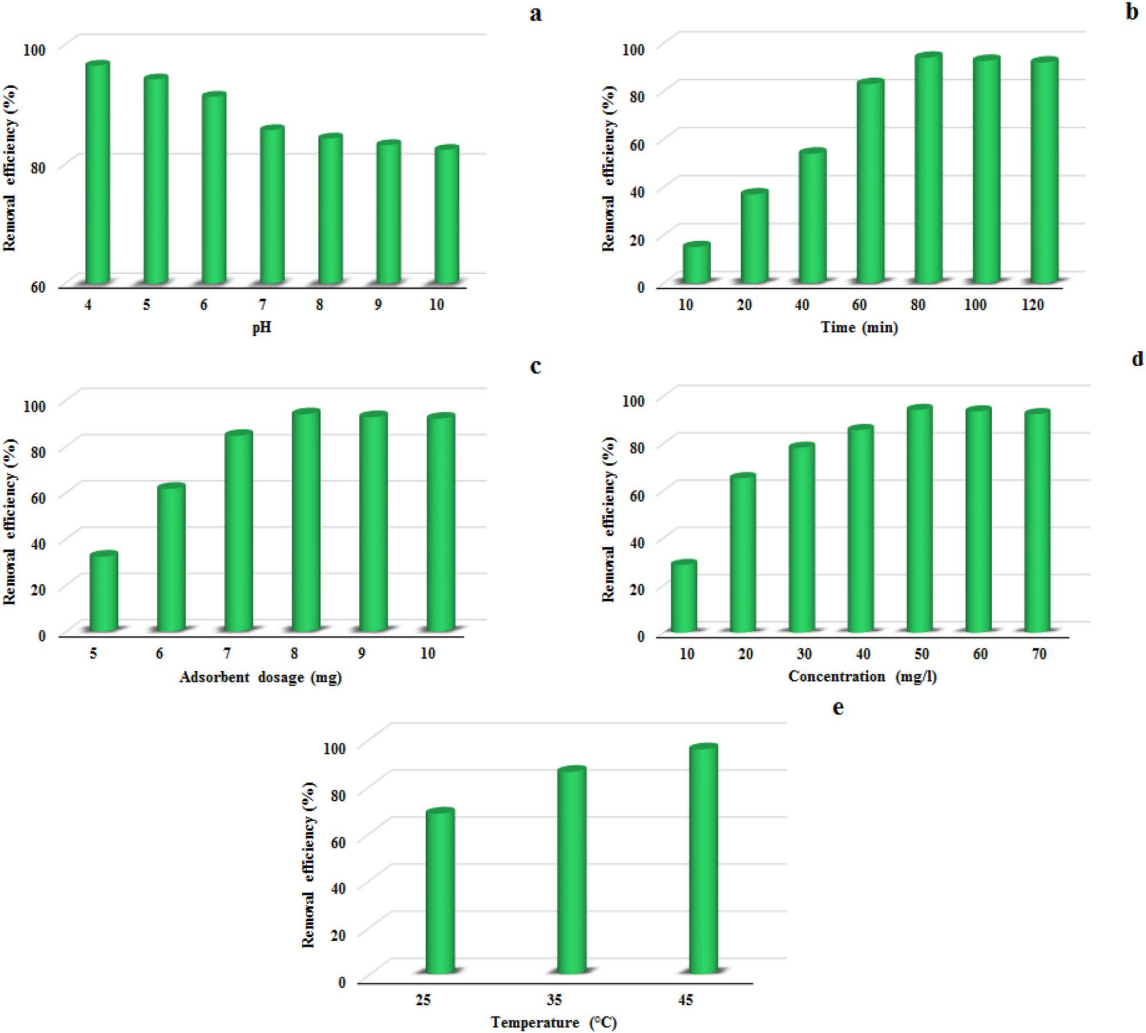
The effect of pH (**a**), contact time (**b**), adsorbent dosage (**c**), methyl red concentration (**d**), and temperature (**e**) on MR adsorption.

**Figure 11:**
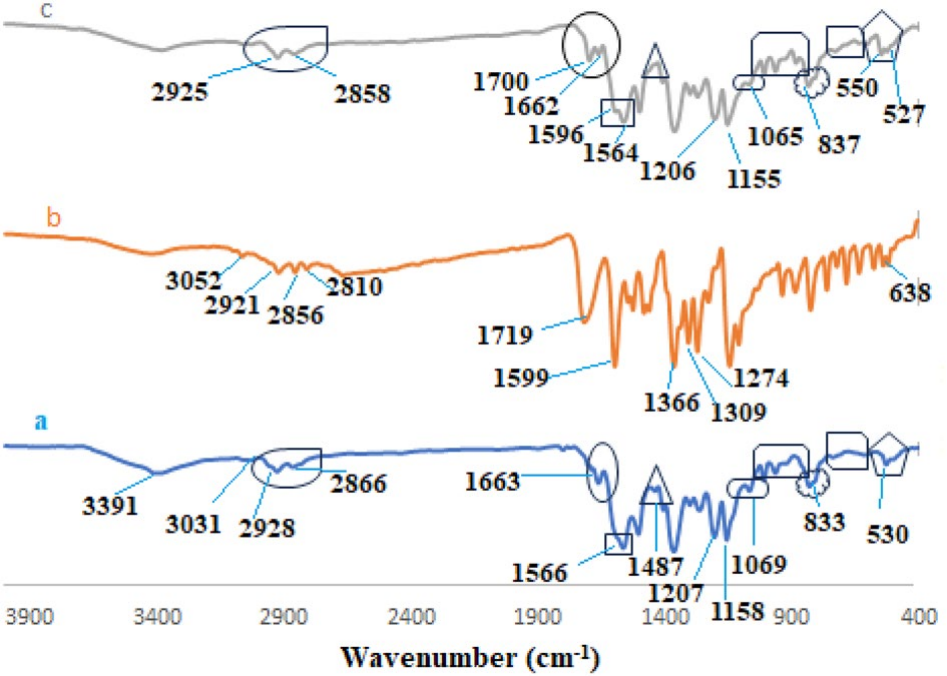
FT-IR spectra of TC-POP (**a**), methyl red (MR) dye (**b**), TC-POP + MR (**c**).

**Figure 12:**
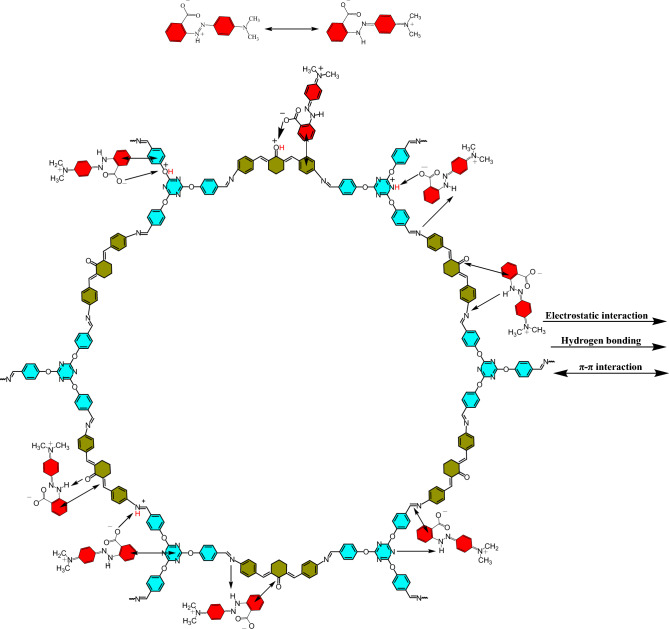
The possible mechanism of MR adsorption on TC-POP.

The pH solution is one of the most sensitive factors in the adsorption process because it can affect the amount of ionization of the organic contaminants, the adsorbent level load, the structure of the pollutants, and the active sites on the adsorbent. Due to the presence of many nitrogen and oxygen atoms as active sites on the adsorbent surface, as well as the presence of functional groups N(CH_3_)_2_, COOH and N=N of methyl red dye, pH plays an important role in the adsorption process. The adsorption of MR on TC-POP was studied at a pH range of 4–10 using 8 mg of TC-POP in 10 mL of 50 mg/L solutions of MR at 45 °C (Fig. 10a). The maximum adsorption of methyl red was observed at pH = 4. Considering the structure of TC-POP adsorbent and methyl red anionic dye, several interaction forces such as the π–π interactions between polymer and dye π orbitals, hydrogen bonding interactions, and electrostatic interactions between anionic dye and polarized sites due to change of pH on the adsorbent surface can be expected. At pH = 4, the active sites on the surface of the adsorbent are protonated, which creates positively charged sites on the adsorbent and strong electrostatic interactions with the anionic dye, as a result, the adsorption capacity increases at this pH. Also, the decrease in adsorption capacity at higher pH can be considered due to the decrease of positive charge sites on the adsorbent. However, the significant adsorption of anionic dye on the adsorbent at high pH is due to other interactions such as π-π and hydrogen bonding between MR and TC-POP. It is worth noting that the surface area of adsorbents has a significant impact on its ability to adsorb pollutants. Materials with a higher surface area have more available sites for pollutant molecules to bind to, resulting in higher adsorption capacity and efficiency. This is why many adsorbents, such as porous organic polymers (POPs), have high surface area and porosity to maximize their adsorption capabilities. Therefore, due to the relatively good Brunauer–Emmett–Teller surface area (S_BET_) (108.27 m^2^ g^−1^) of the synthesized TC-POP, there are many active sites on the polymer, which significantly contributes to the adsorption of methyl red dye. This property, in combination with the other favorable characteristics of the TC-POP, makes it as a promising adsorbent for the removal of methyl red dye from aqueous solutions. The adsorption process of methyl red dye was also investigated using FT-IR spectroscopy. A clear difference between the FT-IR spectra before (TC-POP) and after (TC-POP + MR) adsorption is observed, which supports the dye adsorption process by the TC-POP adsorbent (Fig. 11a–c). The comparison of the two FT-IR spectra and the presence of new absorption bands in the spectrum after the absorption process indicate the formation of a connection between TC-POP and MR. Also, some peaks are disappeared, and the intensity of some peaks reduced or shifted to lower wavelengths^58^. A possible mechanism of MR adsorption on TC-POP is briefly presented in Fig. 12.

### Effect of contact time

As shown in Fig. 10b, the process of methyl red dye adsorption on TC-POP was investigated at different times from 10 to 120 min. Initially, the adsorption process of MR is fast due to the high surface area of the adsorbent and subsequently many active sites of the adsorbent until it reaches equilibrium within 80 min. After that, with increasing contact time, no significant change in adsorption capacity was observed.

### Effect of adsorbent dosage

To investigate the effect of adsorbent amount on MR removal, different adsorbent amounts (5–10 mg) were used, while other parameters such as pH = 4, contact time 80 min, the concentration of MR solution 50 mg/L, and temperature of 45 °C were constant. By increasing the adsorbent dose, the dye removal efficiency also increases, which may be due to the increase in the adsorbent surface and the subsequent increase in the number of active sites for dye adsorption. The dye was rapidly adsorbed up to the adsorbent dose of 8 mg, and no noticeable change in adsorption efficiency was observed at higher concentrations. According to the obtained results, the amount of 8 mg of adsorbent was chosen as the optimal dosage to achieve the maximum dye removal efficiency (Fig. 10c).

### Effect of methyl red dye concentration

The effect of initial MR dye concentration on TC-POP adsorption capacity was examined in the range of 10–70 mg/L with an adsorbent dose of 8 mg at pH = 4 and temperature of 45 °C. As can be seen in Fig. 10d, As the initial dye concentration increases, the adsorption efficiency increases. This tendency continues up to the concentration of 50 mg/L, but at higher concentrations, the adsorption efficiency remains almost constant due to the constant amount of adsorbent and the number of active sites on the adsorbent. Therefore, with the increase of the MR concentration, the active sites of the adsorbent are gradually filled and the polymer is no longer able to adsorb the dye, and with the increase of the dye concentration, the adsorption capacity decreases. So, the optimal initial concentration of 50 mg/L was considered.

### Effect of temperature

Temperature plays an important role in the adsorption process in order to determine whether it is endothermic or exothermic. Therefore, to find the highest adsorption efficiency, the optimum temperature should be obtained. The effect of temperature on the MR adsorption process by the TC-POP adsorbent was investigated at 25, 35, and 45 °C (pH: 4; dose: 8 mg; [MR]: 50 mg/L; time: 80 min). The results can be seen in Fig. 10e. As the temperature increases, the dye adsorption efficiency also increases, which is a sign of the endothermic nature of the adsorption process. The optimal temperature was found to be 45 °C.

### Adsorption isotherms

**Figure 13:**
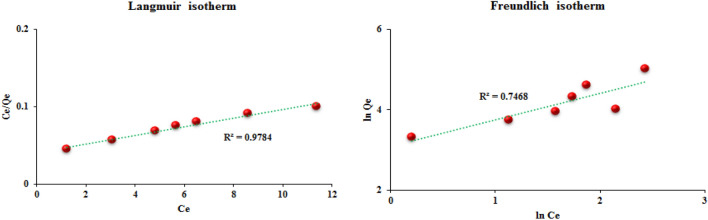
The methyl red adsorption isotherms.

The adsorption isotherm is one of the most important factors for designing the adsorption process system and also describing the reciprocal behavior between the adsorbent and the solute. In addition, the adsorption mechanism can be expressed through adsorption isotherms and is also used to calculate the adsorption capacity of the adsorbent. The Langmuir and Freundlich models are among the most important and widely used models for describing the experimental data of adsorption isotherms. Therefore, in this study, the data obtained from the dye adsorption process on the polymer were analyzed using Langmuir and Freundlich isotherms. In the Langmuir isotherm model, it is assumed that the adsorption of solutes on the adsorbent is carried out in a monolayer, and it also assumes that all the adsorption sites are the same in terms of energy and the adsorbent has a homogeneous structure. Linear Eq. (3) is used to express the Langmuir isotherm model^59^:

3 $$\frac{{C}_{e}}{{Q}_{e}}=\frac{1}{{Q}_{max}.K}+\frac{{C}_{e}}{{Q}_{max}}$$

where C_e_ (mg/L), Q_e_ (mg/g), Q_max_, and K indicate the concentration at equilibrium, the equilibrium adsorption capacity, the maximum adsorption capacity and the Langmuir constants, respectively. In the Freundlich isotherm, which is an experimental model, it is assumed that the solutes are adsorbed in several layers on the adsorbent surface, the adsorbent surface is not uniform and the surfaces have different adsorption power. The linear form of the Freundlich model is described by Eq. (4)^60^.

4 $${LnQ}_{e}= {LnK}_{f}+\frac{1}{n}{LnC}_{e}$$

where n and K_f_ (L/mg) are the Freundlich constants, which indicate the adsorption intensity and adsorption capacity, respectively. The results obtained from the calculation of Langmuir and Freundlich isotherm linear equations are given in Fig. 13 and Table 1. After analyzing the data and the values of the regression coefficient (R^2^), it was found that the adsorption of methyl red by TC-POP follows the Langmuir isotherm model and it can be assumed that the adsorption is performed in a monolayer on the homogeneous adsorbent.

**Table 1:** Adsorption isotherm parameters.

Langmuir model			Freundlich model		
q_max_ (mg g^−1^)	K_L_ (L mg^−1^)	R^2^	K_F_	n	R^2^
178.57	0.137	0.9784	21.812	1.513	0.7468

### Adsorption kinetics

Adsorption kinetic models are used to investigate the contact time required to reach the adsorption equilibrium (speed of the adsorption process) and also to show the interaction mechanism between the adsorbent and the adsorbate. Therefore, in order to better understand the adsorption behavior and mechanism and to evaluate the kinetics of MR adsorption at the TC-POP level, two common kinetic models including pseudo-first-order and pseudo-second-order under optimal conditions at different times were used. Pseudo-first-order kinetic models^61^, Eq. (5), and pseudo-second-order kinetics models^62^, Eq. (6), show that adsorption occurs through physical adsorption and chemical adsorption processes, respectively.

5 $$Ln\left({Q}_{e}-{Q}_{t}\right)=Ln\left({Q}_{e}\right)-t{K}_{1}$$

6 $$\frac{t}{{Q}_{t}}=\frac{1}{{K}_{2}{Q}_{e}^{2}}+\frac{t}{{Q}_{e}}$$

**Figure 14:**
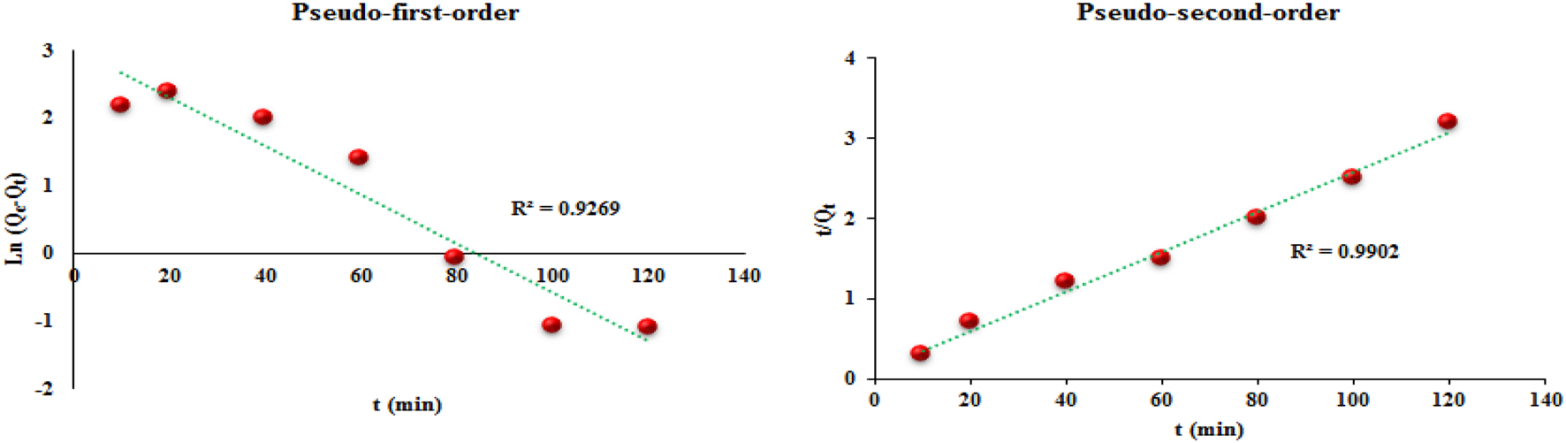
The kinetic models for MR adsorption onto the TC-POP.

In the above equations, Q_e_ (mg g^−1^) is the adsorption capacity at equilibrium, Q_t_ (mg g^−1^) is the adsorption amount at time t (min), K_1_ (L min^−1^), and K_2_ (g mg^–1^ min^–1^) are the pseudo-first-order and pseudo-second-order rate constants, respectively. The results of the adsorption kinetics of MR dye are shown in and Fig. 14 and Table 2. According to the results and correlation coefficients (R^2^), the adsorption of MR by TC-POP is consistent with the pseudo-second-order kinetic model.

**Table 2:** Adsorption kinetic data.

Pseudo-first-order			Pseudo-second-order		
q_e,cal_ (mg g^–1^)	K_1_ (1 min^−1^)	R^2^	q_e,cal_ (mg g^−1^)	K_2_ (g mg, min^−1^)	R^2^
20.52	− 0.00045	0.9269	40.49	0.0015	0.9902

#### Thermodynamic studies

**Figure 15:**
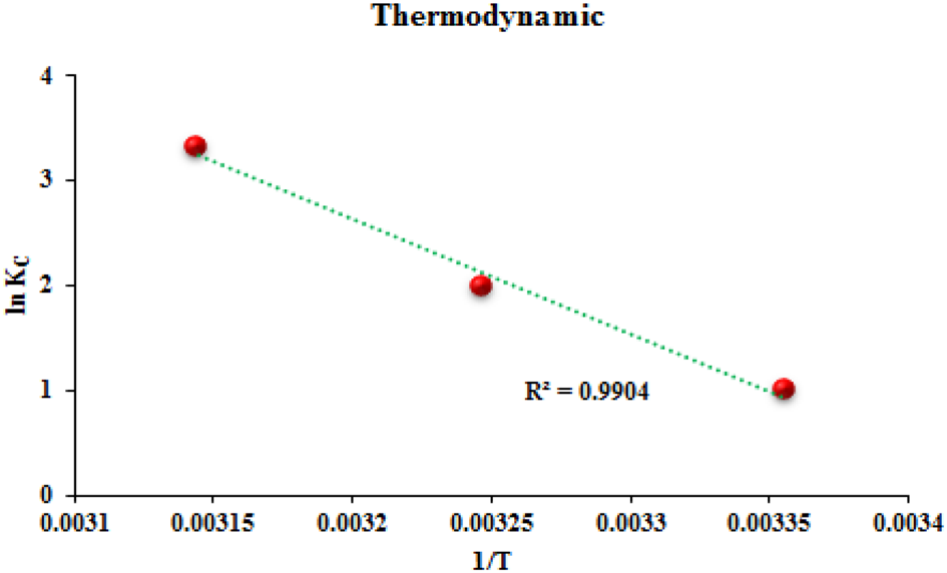
The investigation of thermodynamic properties of adsorption of MR by TC-POP.

The adsorption process can be endothermic or exothermic according to the nature of the adsorbent material and adsorbed molecules. In order to investigate whether MR adsorption on POP is exothermic or endothermic, thermodynamic parameters, Gibbs free energy ΔG° (kJ/mol), entropy ΔS° (J/mol/K), and enthalpy ΔH° (KJ/mol) at several temperatures (298, 308, and 318 K) were calculated by Eqs. (7) and (8)^63^.

7 $${\Delta G}^{^\circ }=-RTLn{K}_{c}$$

8 $$Ln{K}_{c}=\frac{{\Delta H}^{^\circ }}{R.T}+\frac{{\Delta S}^{^\circ }}{R}$$

where R is the gas constant (8.314 J mol^−1^ K^−1^), K_c_ (L mol^−1^) is the equilibrium constant, and T (K) is the adsorption temperature. The obtained thermodynamic parameters are presented in Fig. 15 and Table 3. The positive values of ΔH and ΔS indicate that the adsorption process of MR using TC-POP is endothermic and leads to an increase in the degree of randomness or a decrease in the degree of orderliness of the system. Furthermore, the negative values of ΔG for all temperatures indicate that the adsorption process is spontaneous and possible.

**Table 3:** Thermodynamic parameters.

T (K)	Δ*G*° (KJ mol^−1^)	Δ*H*° (KJ mol^−1^)	Δ*S*° (KJ mol^−1^ K^−1^)
298	− 2.46		
308	− 5.1		
318	− 8.73	90.86	312.63

#### Desorption and reusability studies

**Figure 16:**
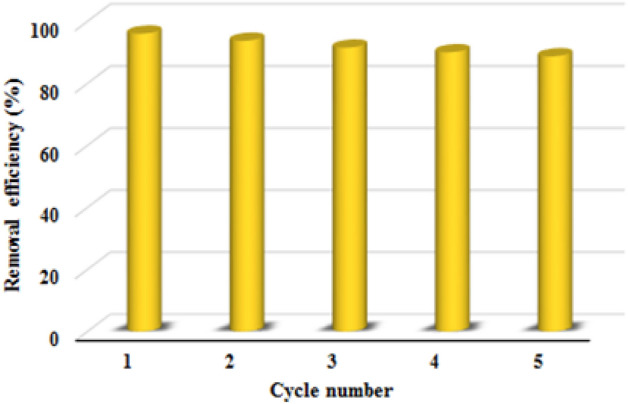
The reusability of the POP for methyl red removal in five consecutive cycles.

One of the vital features of an ideal adsorbent is its ability to be regenerated and reused as well as its high adsorption capacity, which makes its use in practice and industry economical and cost-effective. Therefore, several adsorption–desorption cycles were performed to investigate this feature in the synthesized polymer. To test this feature, 10 mL of MR aqueous solution (50 mg/L) was mixed with 8 mg of TC-POP adsorbent and stirred for 80 min. Then TC-POP was separated from the mixture by centrifugation and washed well with ethylene glycol and deionized water, respectively, dried and used for subsequent adsorption. This process was carried out for five cycles. The results show that the removal efficiency of MR by TC-POP does not change significantly in five cycles and has a slight decrease in each step (Fig. 16). Therefore, the results demonstrate the reusability of TC-POP for MR removal.

The comparison of the maximum adsorption capacity (Q_max_) of the polymer synthesized in this study and other adsorbents for the adsorption of methyl red dye is reported in Table 4. The obtained results show the ideal and acceptable adsorption capacity for TC-POP.

**Table 4:** The comparison of the maximum adsorption capacity of MR on various adsorbents.

Adsorbent	q_m_ (mg g^−1^)	References
Guar gum powder	66.66	^64^
Activated carbon	40.49	^65^
NaOH modified activated carbon	206.08	^66^
Commercial activated charcoal	30	^67^
Coffee residues	76.92	^58^
TC-POP	178.57	This work

## Conclusion

To summarize, a novel triazine and cyclohexanone-based porous organic polymer, (TC-POP), was successfully prepared via Schiff base condensation using 2,4,6-tris-(4-formylphenoxy)1,3,5-triazine (TFPT) and 2,6-bis (4-aminobenzylidene)cyclohexanone. The obtained polymer shows significant physiochemical and thermal resistance, which may be due to its rich π-conjugated structure. It was used as an adsorbent to remove the organic pollutant methyl red dye from an aqueous solution under optimized conditions with excellent adsorption efficiency and acceptable reusability. Adsorption isotherms and adsorption kinetics and thermodynamic parameters were also investigated to obtain the mechanism, speed, and endothermic or exothermic nature of the adsorption process. Analysis of the results showed that the adsorption of MR on TC-POP has pseudo-second-order kinetics and a Langmuir adsorption model. Also, the calculated thermodynamic parameters show that the adsorption process is endothermic and spontaneous. The existence of oxygen and nitrogen atoms as well as aromatic rings in the polymer structure causes strong π–π interactions and electrostatic, and hydrogen bonding between it and the dye and removal of the dye from the water. After 5 times of recycling in the adsorption process, the synthesized TC-POP showed a good performance and the adsorption efficiency was slightly changed. These results can indicate the capabilities of this polymer as a reliable and ideal adsorbent for the adsorption of methyl red dye and toxic pollutants from water and wastewater.

### Supplementary Information

Supplementary Information.

## Data Availability

The authors confirm all data generated and analyzed during this study are available in the article and its supplementary material.
