# Supplementary material for: Synthesis of a novel porous organic polymer containing triazine and cyclohexanone rings as an efficient methyl red adsorbent from aqueous solutions
Source: Sci Rep. 2023 Aug 10;13:12962. doi: 10.1038/s41598-023-40274-7 (PMC10415288; doi:10.1038/s41598-023-40274-7)
Supplement: Supplementary file 1 — Supplementary Information. [file 41598_2023_40274_MOESM1_ESM.docx]

**Supporting Information**

**Synthesis of a novel porous organic polymer containing triazine and cyclohexanone rings as an efficient methyl red adsorbent from aqueous solutions**

Javad Ghanbari^1^, Akbar Mobinikhaledi^1,2*^

^1^Department of Chemistry, Faculty of Science, Arak University, Arak, 38156-88138, Iran

^2^Institute of Nanosciences and Nanotechnology, Arak University, Arak, Iran

*Corresponding author: akbar_mobini@yahoo.com

**2,4,6-tris-(4-formylphenoxy)1,3,5-triazine (TFPT)**

White solid, yield (89%), mp (172-174 °C); IR (KBr) (ῡ_max_, cm^-1^): 1213 (C-O), 1365 (C-N), 1568 (C=N),1598 (C=C) 1701 (C=O), 2741-2834 (C-H aldehyde), 3071, 3103 (C-H aromatic).

^1^H-NMR (300 MHZ, CDCl_3_, ppm): δ; 7.33 (d, 6H, Ar-H, *J*= 6.0 Hz), 7.94 (d, 6H, Ar-H, *J*= 6.0 Hz), 10.01 (s, 3H, COH).

^13^C-NMR (75 MHz, CDCl_3_, ppm): δ; 190.6 (3C=O aldehyde), 173.2 (3C=N triazine),155.6 (3C aromatic), 134.4 (3C aromatic), 131.3 (6C aromatic), 122.2 (6C aromatic).


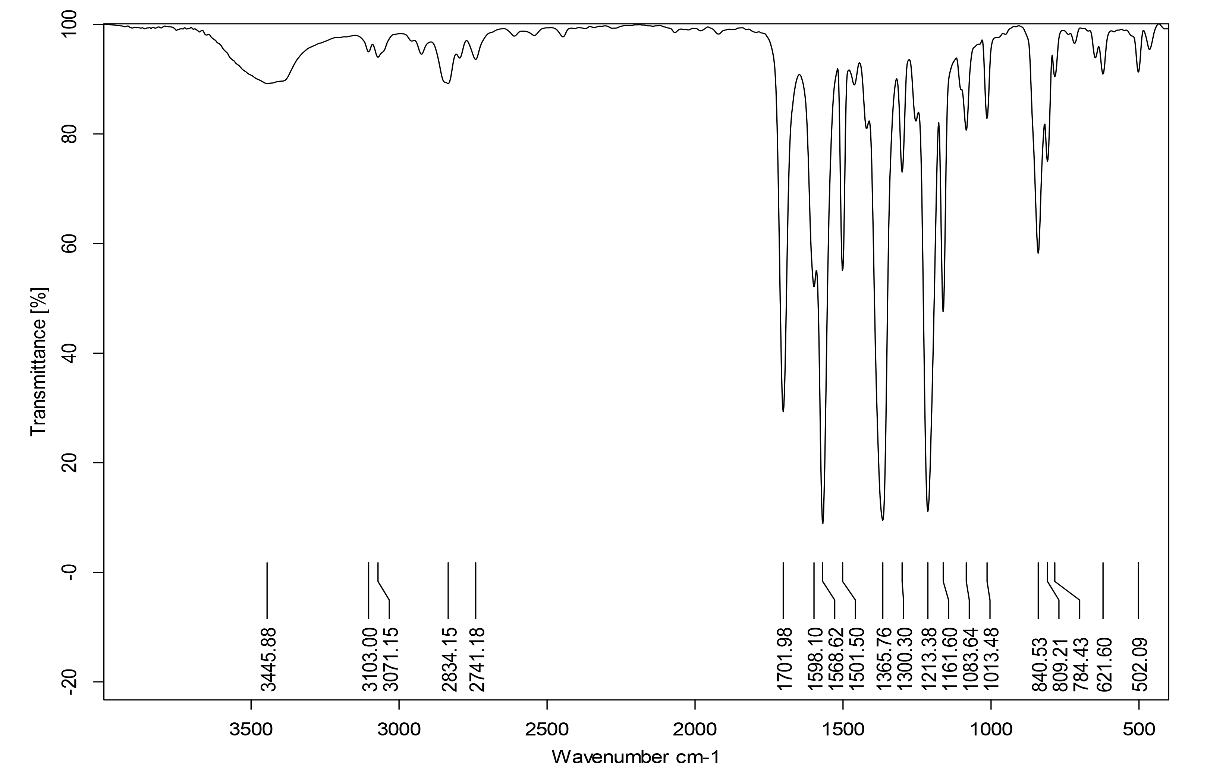


**Figure S1.** FT-IR spectrum of TFPT**.**


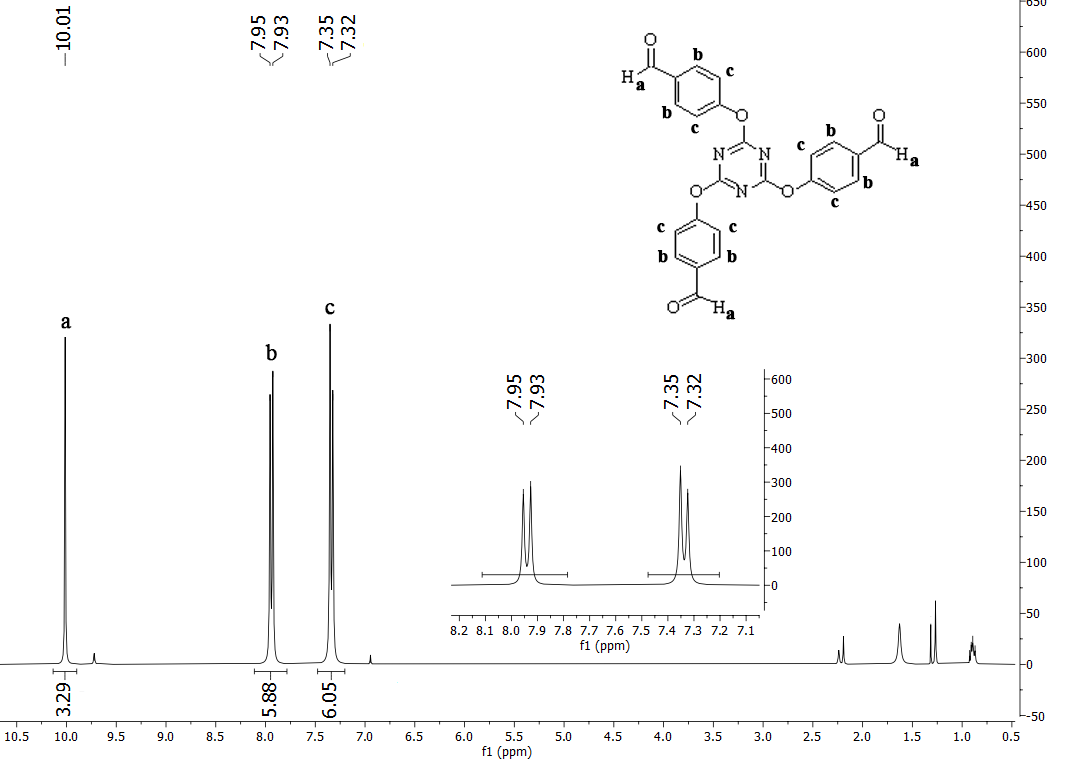


**Figure S2.** ^1^H-NMR spectrum of TFPT**.**


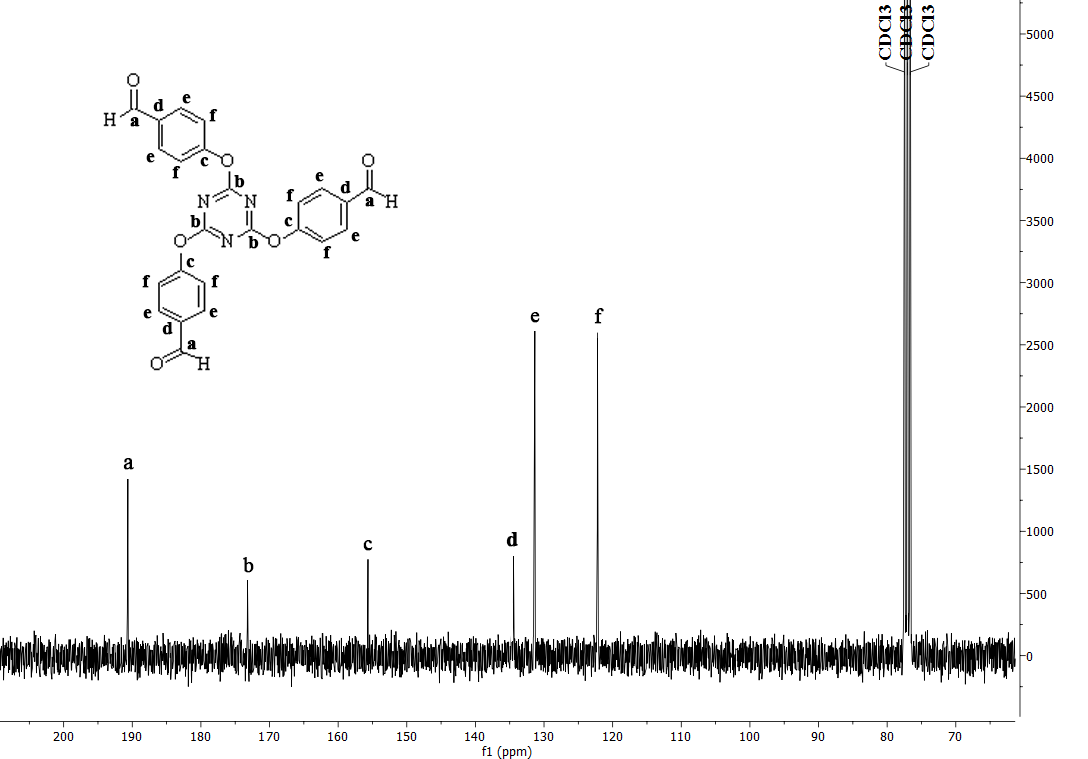


**Figure S3.** ^13^C-NMR spectrum of TFPT**.**

Yellow solid, yield (93%), mp (209-211 °C); IR (KBr) (ῡ_max_, cm^-1^): 3103 (w), 2922 (w), 1669 (s), 1588 (s), 1513 (s), 1342 (s), 1300 (m), 1265 (s), 1160 (w), 1140 (w), 1107 (m), 1011 (m), 965 (m), 922 (m), 855 (s), 800 (m), 715 (m), 513 (w).


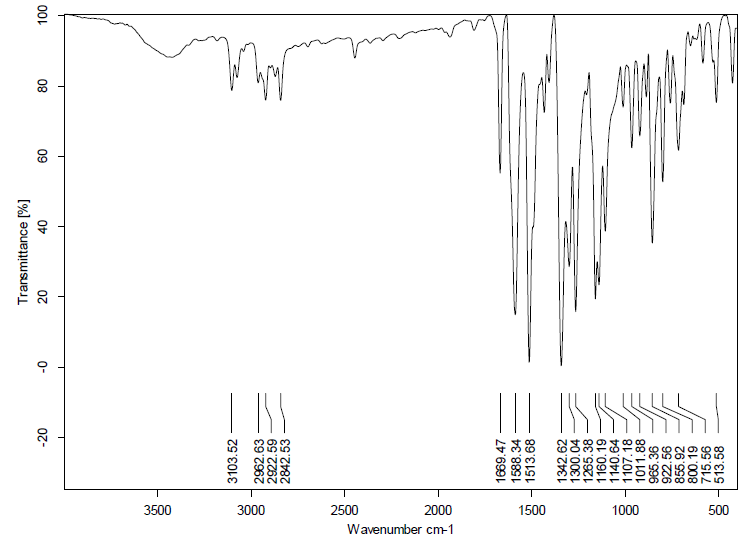


**Figure S4.**  FT-IR spectrum of 2,6-bis(4-nitrobenzylidene)cyclohexanone.

light brown solid, yield (90%), mp (290-292 °C); IR (KBr) (ῡ_max_, cm^-1^): 3349 (m), 3225 (m), 3050 (w), 2926 (w), 1628 (m), 1580 (s), 1509 (s), 1433 (s), 1344 (w), 1285 (s), 1204 (w), 1160 (s), 1067 (w), 966 (s), 833 (s), 740 (w), 711 (w), 530 (s), 493 (s).

^1^H-NMR (300 MHZ, DMSO-d_6_, ppm): δ; 7.47 (s, 2H, a), 7.26-7.28 (d, 4H, *J*= 6.0 Hz, b), 6.60-6.62 (d, 4H, *J*= 6.0 Hz, c), 5.70 (s, 4H, d), 2.80-2.84 (m, 4H, e), 1.68-1.72 (m, 2H, f).

^13^C-NMR (75 MHz, DMSO-d_6_, ppm): δ; 188.4 (a), 150.4 (b), 136.9 (c), 132.9 (d), 131.4 (e), 123.4 (f), 113.9 (g), 28.6 (h), 23.1 (i).


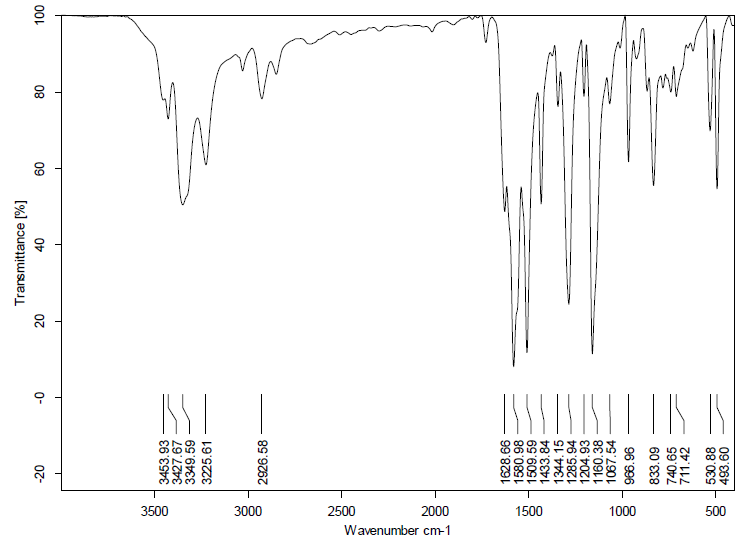


**Figure S5.** FT-IR spectrum of 2,6-bis(4-aminobenzylidene)cyclohexanone.

**
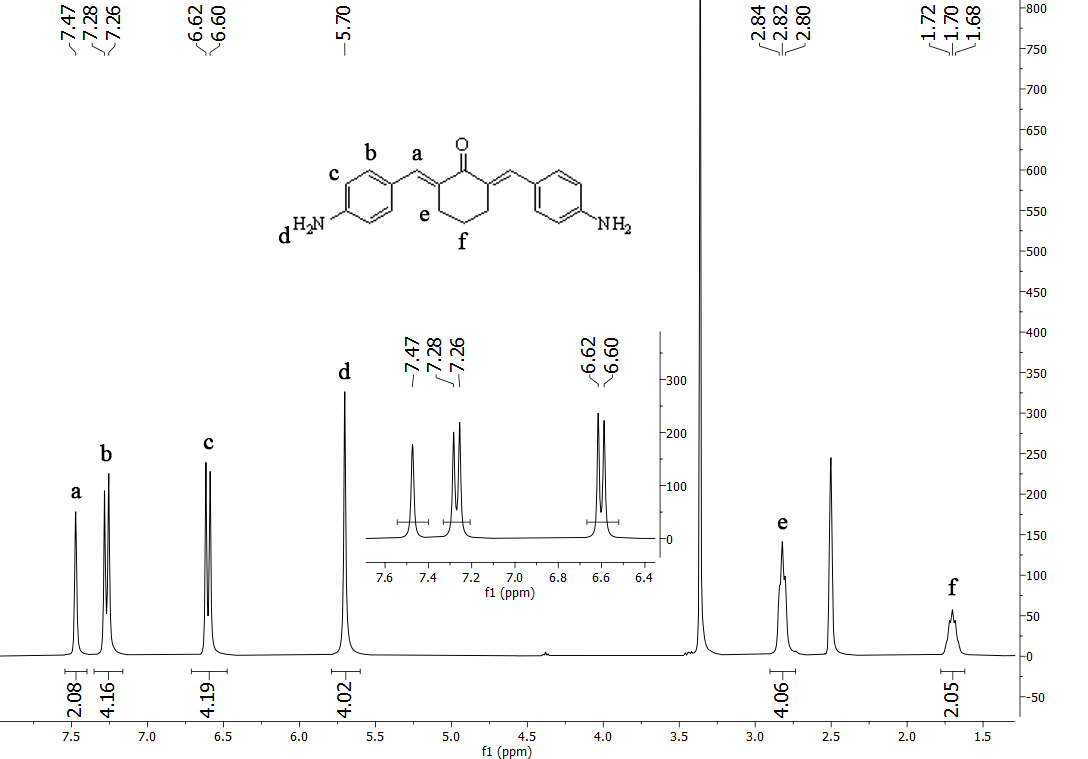
**

**Figure S6.** ^1^H-NMR spectrum of 2,6 bis(4-aminobenzylidene)cyclohexanone**.**


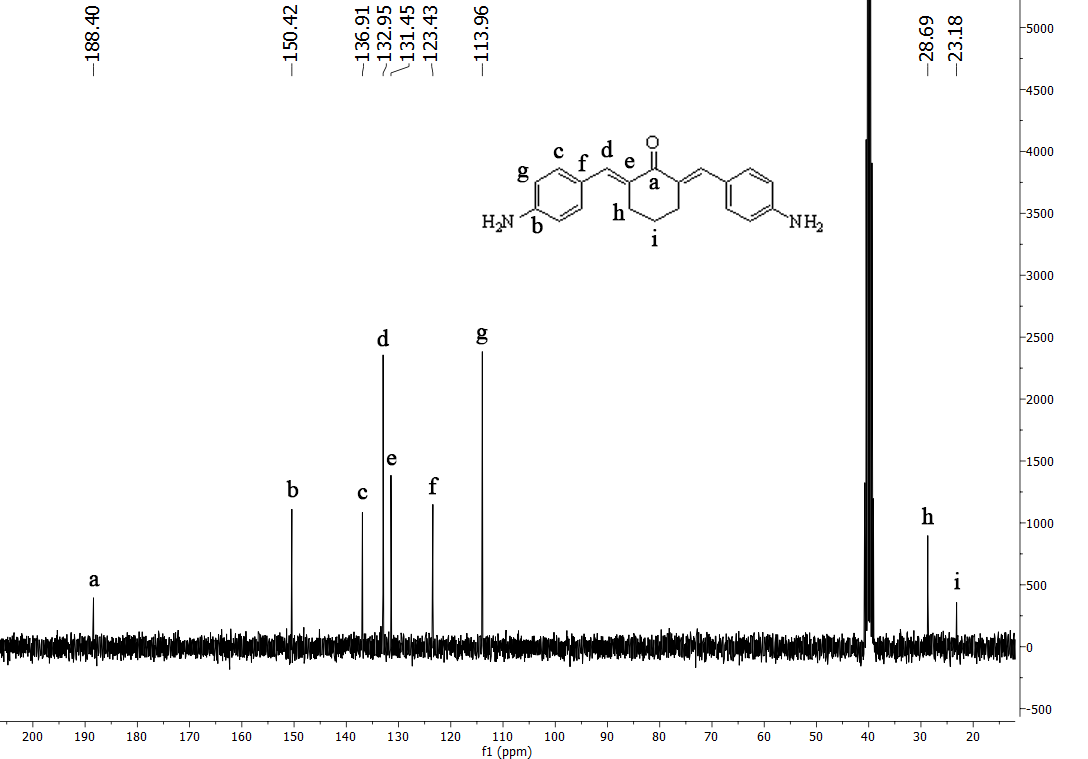


**Figure S7.** ^13^C-NMR spectrum of 2,6 bis(4-aminobenzylidene)cyclohexanone**.**
